# Biochemical diagnosis of Sanfilippo disorder types A and B

**DOI:** 10.1186/s43141-023-00586-7

**Published:** 2023-11-10

**Authors:** Soha S. Nosier, Seham M. S. El Nakeeb, Mona M. Ibrahim, Mona El-Gammal, Ekram M. Fateen

**Affiliations:** 1 Biochemical Genetics Department, Human Genetic and Genome Research Institute, National Research Centre, Cairo, Egypt; 2 Medical Biochemistry Department, Faculty of Medicine (for Girls), Al-Azhar University, Cairo, Egypt; 3 Clinical Genetics Department, Human Genetic and Genome Research Institute, National Research Centre, Cairo, Egypt

**Keywords:** Mucopolysaccharidosis, Sanfilippo disorder, *N*-sulphoglucosamine sulphohydrolase, *N*-alpha-acetylglucosaminidase enzymes

## Abstract

**Background:**

One of the 11 recognized mucopolysaccharidosis (MPS) diseases is Sanfilippo. It is autosomal recessive in its mode of transmission. There are four subtypes of Sanfilippo (A, B, C, and D). The most worldwide prevalent subtypes of mucopolysaccharidosis type III (MPS III) are A and B followed by C and D subtypes. To estimate the frequency of MPS IIIA among MPS III patients, we diagnose and compare their clinical features with those of MPS IIIB and also compare the prevalence of MPS IIIB versus MPS IIIA among diagnosed cases at the Biochemical Genetic Department at NRC. For every case that was referred, the quantitative determination of urine Glycosaminoglycans (GAGs) was assessed. Two-dimensional electrophoresis (2DE) of GAGs extracted from urine was performed on all cases with high urinary GAG levels. Both *N*-sulphoglucosamine sulphohydrolase (MPS IIIA) and *N*-alpha-acetylglucosaminidase (MPS IIIB) enzyme activity were determined fluorometrically.

**Results:**

From November 2019 to May 2022, 535 cases were referred to the National Research Centre’s Biochemical Genetics Department. 233 (43%) MPS cases were diagnosed with high urinary GAG levels for their ages. 73 (31.3%) MPS III cases were diagnosed by 2DE out of the 233 MPS cases. Plasma *N*-alpha-acetylglucosaminidase enzyme assay was insufficient in 36 (49.3%) patients (Sanfilippo type B), while *N*-sulphoglucosamine sulphohydrolase enzyme activity was deficient in 15 (20.6%) patients. The other 22 (30.1%) patients are either Sanfilippo type C or D.

**Conclusion:**

*N*-sulphoglucosamine sulphohydrolase enzyme activity was measured for the first time in Egypt. Thirty-one percent of all diagnosed MPS cases during the last 3 years were MPS type III, making Sanfilippo the most common MPS type among the referred cases to our Biochemical Genetics Department. MPS IIIA accounts for 20.6% of MPSIII cases in this study. Still, MPS type IIIB is the commonest type among diagnosed patients.

**Supplementary Information:**

The online version contains supplementary material available at 10.1186/s43141-023-00586-7.

## Background

Sanfilippo syndrome, MPS III is one of the lysosomal storage disorders which is a neurodegenerative disease. It is caused by enzymatic defects in heparan sulphate (HS) catabolism. Sanfilippo has four subtypes A, B, C, and D; each has its own genetic mutation that results in *N*-sulphoglucosamine sulphohydrolase, α-*N*-acetylglucosaminidase, α-glucosaminide-*N*-Ac-transferase, and glucosamine-*N*-sulphatase enzyme deficiency, respectively. The most common subtypes are MPSIIIA and B [1]. Any of these enzymes’ deficiencies cause subsequent HS accumulation in lysosomes of affected tissues and organs [2].

Neurological manifestations such as cognitive decline, hyperactivity, aggressiveness, sleep disturbances, and/or epilepsy are the main symptoms of Sanfilippo. These are associated with significant abnormalities of the central nervous system (CNS), including neurological manifestations, brain atrophy, and spinal cord compression [3]. Delayed milestones and neurological manifestations as hyperactive behaviors are the first symptoms of the disease and start at the age of 2 or 3 years [4].

The first laboratory investigations of MPS III after clinical suspicion are quantitative and qualitative 2DE GAG measurement in urine that demonstrates excessive HS excretion [5]. Specific enzyme measurement in plasma or leukocytes is the 2nd step in diagnosis; *N*-sulphoglucosamine sulphohydrolase (SGSH, EC 3.10.1.1) for MPS IIIA, *N*-acetyl-α-glucosaminidase (NAGLU, EC 3.2.1.50) for MPS IIIB, heparan-α-glucosaminide *N*-acetyl transferase (EC 2.3.1.78) for MPS IIIC, and *N*-acetyl glucosamine 6-sulphatase (EC 3.1.6.14) for MPS IIID. The final step is molecular variant identification for the gene encoding the deficient enzyme [6].

Signs and symptoms of Sanfilippo disorder progression are variable partially due to the amount of residual enzyme activity dictated by the molecular variant. The clinical course is affected by genotype which in some times not as expected may be due to epigenetic factors [7].

The neuro-pathological manifestations (main feature) of Sanfilippo are due to Lysosomal HS accumulation in the brain [8]. In this study, we measured the enzyme activity for Sanfilippo A and B. MPS III A was 20.6% and MPS III B 49.3%, while the rest cases, 30.1% were suspected to be MPS III C or D. This study should establish Sanfilippo type A enzyme assay method and try to give impact about the percent of these cases in Egypt in relation to Sanfilippo B, C, and D.

Using enzyme replacement therapy is under trial but without medical approval till now. Supportive treatments are only to delay multisystem complications, but curative therapies that prevent heparan sulfate accumulation are not found till now [9].

### Subjects and methods

#### Subjects and ethics

Five hundred fifty-three cases were referred to the Biochemical Genetic Department, National Research Centre, during the period from November 2019 to May 2022. Their age ranged between 9 months to 17 years. A control group of matching age and sex to the study group was used in the study. All participants’ parents gave written informed consent after a full explanation of the study. The ethical approval was obtained from the Medical Ethical Committee at the NRC and the Faculty of Medicine, Al Azhar University (Girls), Cairo, Egypt (The Faculty of Medicine, Al Azhar University (Girls), Approval no. 201910199).

Complete history taking and pedigree construction were recorded for each patient.

#### Methods

Mentioned in detail with supplementary data (Additional file 1).

### Statistical analysis

All data were coded and organized, and then, SPSS program version 16 was used for statistical analysis. Shapiro–Wilk test was used to test the data normality and if it is normally distributed also to describe the nonparametric data in numbers. Differences between groups were analyzed for significance using a one-way ANOVA with tests of homogeneity of variances. Correlations were performed using case summaries, chi-squared test of homogeneity, and independent sample effect sizes with Levene’s test for equality of variances. The comparison between the two variables was calculated by Pearson’s rho coefficient(r). The *p* value < 0.05 was considered significant.

## Results

According to enzyme assay results, 36 (49.3%) cases had deficient alpha-*N*-acetylglucosaminidase enzyme activity, 15 (20.6%) cases had deficient *N*-sulphoglucosamine sulphohydrolase enzyme, and 22 (30.1%) patients had a normal level of both enzymes. These 22 cases are diagnosed provisionally as MPS III C or D for further confirmation.

The study included 535 subjects referred to the Biochemical Genetic Department at Human Genetics and Genome Research Institute, National Research Centre. They included 302 (56.4%) males and 233 (43.6%) females.

The age range within 535 cases was from 1 month to 20 years. The mean age of diagnosis in 48/73 (65.7%) MPS III cases was 5 years, 7/73 (9.6%) cases were early diagnosed at the age of 3 to 12 months, and 18 cases were diagnosed at the age of 6 to 17 years.

Four hundred thirty (80.4%) cases had consanguineous parents while 105 (19.6%) cases had non-consanguineous parents. The consanguinity rate in the present study was 64/73 (87.7%) in MPS III patients, 32/36 (88.9%) cases in MPS IIIB patients, 19/22 (86.4%) cases in MPS IIIC or D patients, and 13/15 (86.7%) cases in MPS IIIA patients (Fig. 2).

The 233 MPS diagnosed cases were 143 (61.4%) male and 90 (38.6%) female, and they were 2 months to 17 years old. One hundred eighty-three (78.5%) MPS patients had positive parental consanguinity against 50 (21.5%) patients with non-consanguineous parents.

**Fig. 1:**
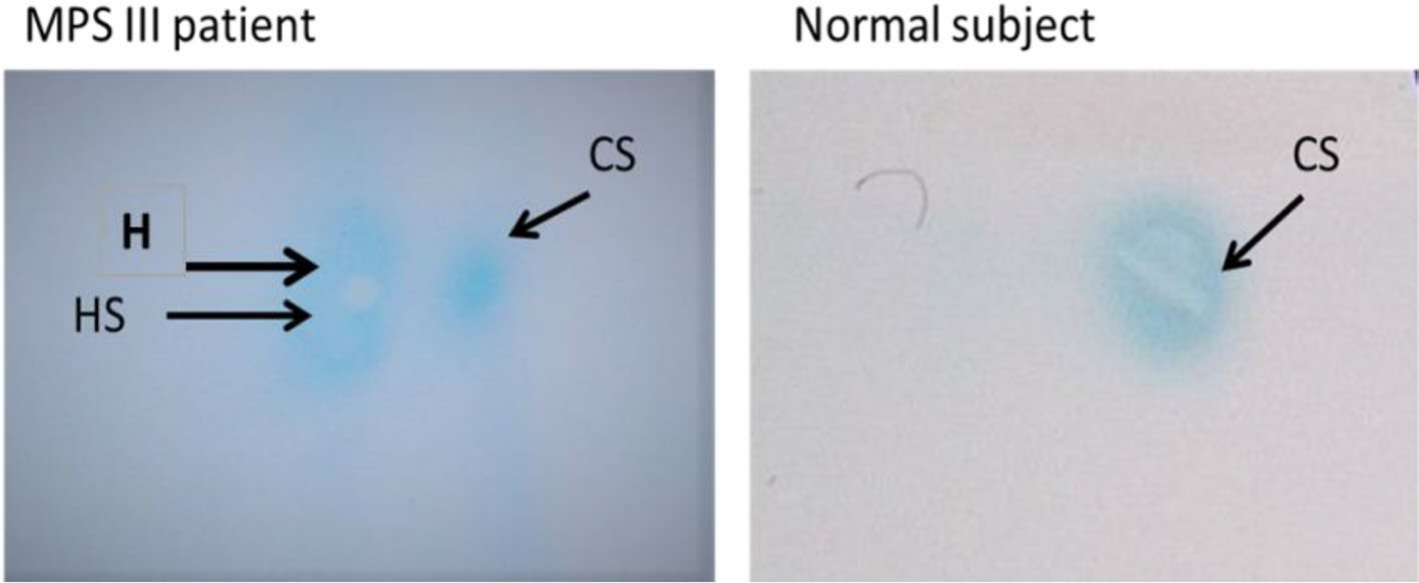
Two-dimensional electrophoresis pattern in normal and Sanfilippo subjects. CS chondroitin sulfate, H heparan, and HS heparan sulfate

Out of 233 MPS cases, 73 MPS III cases were diagnosed due to the presence of heparan and heparan sulfate spots in their 2DE (Fig. 1). MPS III cases included 48 (65.8%) males and 25 (34.2%) females with age ranging from 3 months to 17 years. Sixty-four cases (87.7%) had consanguineous parents, while 9 cases (12.3%) had negative consanguineous marriages.

### Clinical findings

All the 535 referred cases were suspected to have MPS. Clinical examination of MPS III cases showed signs and symptoms represented in Figs. 3 and 4.

Manifestations of hyperactivity were found in 86.7% of MPS IIIA cases, 80.6% of MPS IIIB cases, and 59.1% of MPS IIIC or D cases (Fig. 4).

Nineteen patients (26%), 11 males, and 8 females had general ear problems (Table 2). Out of the 19 cases, 5 (26.3%) cases had MPS IIIA, 11 (57.9%) cases had MPS IIIB, and 3 (15.8%) cases had MPS IIIC or D (Fig. 4).

Cardiac anomalies were detected in 5 cases, 3 cases with MPS type IIIA and 2 cases with MPS IIIB (Figs. 3 and 4). Four cases out of 5 had mitral regurgitation, and one MPS IIIA case had aortic regurgitation.

It was found that 5 years are the mean age of diagnosis of 73 MPS III. Thirty-six patients were < 5 years (from 3 months to 4 years and 6 months), while 27 patients were > 5 years (from 4 and 6/12 to 17 years) and ten patients were 5 years old at the time of diagnosis.

### Biochemical finding

#### Urinary glycosaminoglycans levels

Glycosaminoglycans were measured in the urine of all 535 MPS suspected cases. GAG level was high in 242 cases in relation to their age. Our reference values for urinary normal GAG levels according to Dong et al. [10] are shown in Table 1.

**Table 1:** The mean values of urinary glycosaminoglycans depend on their age [10]

Age	Mean (SD)
0–5 months	33.9 (9.2)
6–12 months	23.3 (4.1)
1 year	19.5 (5.2)
2–3 years	14.5 (3.4)
4–5 years	11 (1.7)
6–7 years	9.3 (1.8)
8–9 years	8.4 (1.6)
10–14 years	7 (1.8)
15–19 years	4.1 (1.3)
> 20 years	3.3 (0.9)

Urinary GAG levels of 73 MPS III cases are presented in Table 2. According to age, the studied cases were classified into 3 groups, group I (0–12 months) which included 7 cases, group II (1–5 years) which included 38 cases, and group III (> 5 years) which included 28 cases. Nine months were the mean age among group I, 3 years among group II, and 8 years among group III. Group I showed 48.8 mg/mmol of creatinine as the mean level of urinary GAGs, while group II and III urinary GAG mean levels were 47.4 and 27 mg/mmol of creatinine, respectively. Urinary GAG levels (means ± SD) of MPS IIIA and MPS IIIB cases were 47.5 ± 0.15 and 25.6 ± 0.27 mg/mmol of creatinine, respectively, as shown in Table 3.

**Table 2:** The mean levels of total urinary glycosaminoglycans (GAGs) among MPS III patients according to their age group

Age group	Number of patients, *n* = 73 (%)	Mean age	Urinary GAGs (mg/mmol creatinine) (mean ± SD)
Group I (0–12 months)	7 (9.6%)	9 months	48.8 (± 0.23) *
Group II (1–5 years)	38 (52%)	3 years	47.4 (± 0.21) *
Group III (> 5 years)	28 (38.4%)	8 years	27 (± 0.11) *

The asterisk symbol “*” means a significant *p* value < 0.05 in comparison against controls

**Table 3:** The mean urinary GAG level of MPS IIIA patients in relation to that of MPS IIIB patients

MPS III A GAGs (mg/mmol creatinine) Mean ± SD	MPS III B GAGs (mg/mmol creatinine) Mean ± SD	*P* value
**25.6 ± 0.27**	47.5 ± 0.15	< 0.04*

The asterisk symbol “*” means a significant *p* value < 0.05

*SD* standard deviation, *GAGs* glycosaminoglycans

#### Two-dimensional electrophoresis in urine

Two-dimensional electrophoretic separation of urinary GAGs was done on 242 cases that showed high levels of urinary GAGs. Two hundred thirty-three cases showed abnormal patterns of MPS types, but 73 patients showed heparan and heparan sulfate spots (MPS III pattern) (Fig. 1) and nine cases with high urinary GAGs and normal 2DE pattern.

##### Alpha-N-acetylglucosaminidase enzyme assay

Alpha-*N*-glucosaminidase enzyme activity was measured in all 73 MPS III after being diagnosed by 2DE. Thirty-six patients had low enzyme activity which confirmed the diagnosis of MPS IIIB (Table 4).

**Table 4:** Comparison between leukocytes alpha-*N*-acetylglucosaminidase enzyme activities in different MPS III types

Alpha-*N*-acetylglucosaminidase enzyme activity	Normal range	Number of cases (*n* = 73)	Mean ± SD nmol/mg prot/hr	*P* value
In MPS III A cases	10–45 nmol/mg prot/h	15	22.31 ± 0.12	<0.001*
In MPS III B cases		36	0.24 ± 0.01	
In MPS III C or D cases		22	15.33 ± 0.11	

Significant *p* value $<$ 0.05, *Significance *p* value

Alpha-*N*-acetylglucosaminidase enzyme activity normal range = 10–45 nmol/mg prot/h

#### N-sulphoglucosamine sulphohydrolase enzyme assay

*N*-sulphoglucosamine sulphohydrolase enzyme activity was measured in the 37 cases that showed normal *α*-*N*-acetylglucosaminidase enzyme activity. Fifteen (40%) patients had low enzyme activity (Table 5). The remaining 22 cases with normal *α*-*N*-acetylglucosaminidase (Table 4) and *N*-sulphoglucosamine sulphohydrolase (Table 5) enzyme levels were accordingly diagnosed as either MPS IIIC or D for further enzymatic study.

**Table 5:** *N*-sulphoglucosamine sulphohydrolase enzyme activity in leukocytes

	**Normal range**	**Number of cases**	**Mean ± SD** **nmol/mg prot/17 h**	***P* value**
MPS III A cases	3.4–42.6 nmol/mg prot/17 h	15	0.25 ± 0.19	0.008*
MPS III C or D		22	12.8 ± 0.23	

Significant *P* value $<$ 0.05, *Significance *p* value

*N*-sulphoglucosamine sulphohydrolase enzyme normal range = 3.4–42.6 nmol/mg prot/17 h

Sanfilippo A cases showed almost the same parental consanguinity as that of the 73 Sanfilippo cases as 13 (86.7%) MPS III cases had consanguineous parents. Comparison within three diagnosed MPS III subtypes (A, B, and C or D) according to the mean level of *α*-*N*-acetylglucosaminidase enzyme assay showed significant differences as represented in Fig. 5.

#### IQ test

IQ test was done on 50/73 (68.5%) MPS III patients, and the average level was (< 18 months). Out of the 23 cases who did not undergo IQ evaluation, 8 cases stopped following up, 6 cases had bone marrow transplants, and 9 cases could not complete the test due to hyperactivity or severe regression stage of the disease. 11/50 (22%) patients were MPS IIIA, 23/50 (46%) MPS IIIB, and 16/50 (32%) MPS III types C or D (Fig. 4).

## Discussion

Sanfilippo disease is an autosomal recessive disorder caused by a genetic mutation in one of the responsible four enzymes for heparan sulfate degradation. Accordingly, there are 4 subtypes of MPS III A, B, C, and D. Accumulated heparan and heparan sulfate in various body organs especially the brain results in MPS III signs and symptoms [11].

In the present study, MPS-suspected subjects were fewer than those investigated in Fateen et al.’s studies (1448 and 1294), respectively. This could be due to a short study period (3 years versus 6 and 11 years, respectively). In these studies, MPS III cases were 28% in Fateen et al. and 15% in Fateen et al., while it was higher in this study. Another earlier study has discussed the Egyptian inborn errors of metabolism (IEM) over 15 years of experience and lysosomal storage diseases (LSD) had the highest percent of diagnosed cases (69.4%) of all IEM. Out of LSD cases, 48.9% had one of the MPS disorders and 17.3% had MPS III [12].

All these studies proved that mucopolysaccharidosis disorders are the commonest subtype of LSD in Egypt, and of all MPS types, MPS III had the highest incidence. This was explained by a high rate of consanguineous marriages in Egypt [13]. Also, late or misdiagnosed cases cause the absence of genetic counseling for affected families that allow the repetition of many cases in the same family.

A Tunisian MPS study in the period of 1970–2005 showed 96/132 MPS cases and 30 (31.2%) MPS III making it the most common MPS type in Tunisia [13]. Another Saudi Arabia study detected 49/189 (25.9%) inborn error of metabolism (IEM) cases, 15/49 (30.6%) MPS cases, and 2/15 (13.3%) MPS III cases [14]. In Egypt, MPS has a higher incidence due to the larger population number and also due to the presence of diagnostic facilities at our Biochemical Genetic Department lab for more than two decades.

**Fig. 2:**
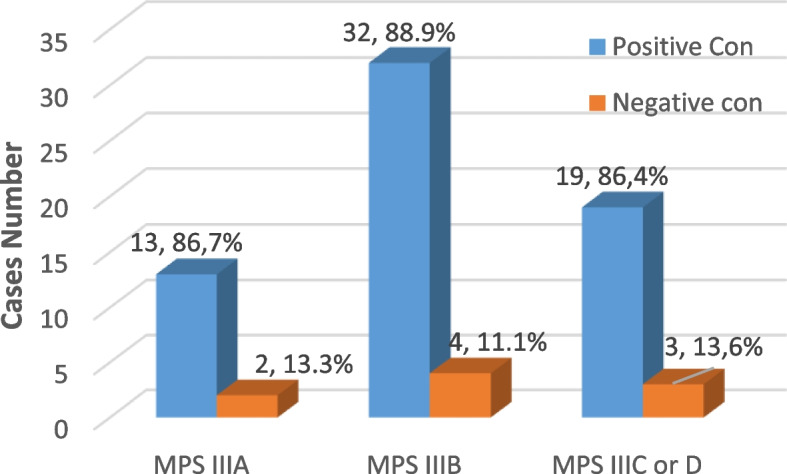
Consanguinity percent among parents of MPS IIIA, B, and C or D patients

The high rate of consanguinity within Arab populations may be due to deeply rooted cultural traditions [15–17]. The consanguinity rate in the present study (Fig. 2) matches the latest Fateen et al., study consanguinity rate of 76%, which is very high helping the accumulation of deleterious genes in the families [15, 17]. These shows the importance of awareness campaigns for the population and the affected families’ genetic counseling.

Male to female ratio in MPS cases was 1.9:1 which is common among Arab populations especially in rural areas. This agrees with other studies that recorded high ratios as in Fateen et al. study the male-to-female ratio was 1.7:1, 1.4:1 in Fateen et al. and 1.2:1 in Ben Turkia et al. study [12, 13, 15].

In our study, the mean age of diagnosis of MPS III cases was 5 years and the 7 early diagnosed cases as they were already siblings of affected patients. The late diagnosis of 18 cases may be because they were the first cases diagnosed in the family or they were attenuated phenotypes of Sanfilippo. In relation to Fateen et al., they also noticed a broad range in the age of onset and diagnosis of MPS cases (1 day to 20 years), while Rouse et al. showed that the 1st phase of MPS III may start from 2 to 6 years [12, 18]. Rouse et al. said that this delay in diagnosis usually accompanies MPS III cases due to a general lack of knowledge of MPS III disease [18].

IQ test (Stanford-Binet IV Intelligence Test) done on MPS III patients’ average level was (< 18 months) (Fig. 4). In the study of Valester et al., 66 MPS III cases had low IQ tests, 27 cases had an IQ level below 3 months, and 39 cases with an IQ level from 6 months to 6 and half years. They included 32/66–48.5% patients with MPS IIIA, 22/66–33.3% patients with MPS IIIB, and 12/66–18.2% patients with MPS IIIC. Their age ranged from 1 to 77 years old while in the present study, patients’ age ranged from 9 months to 17 years. IQ levels among Valester et al. study were higher than that in our study (< 18 months). Both differences in IQ level and age range could be explained by ethnic and mutational variations that affect disease phenotype and subsequently the age of onset [7].

In the present study, aggressive behaviors and hyperactivity were often found in MPS IIIA cases (86.7%) followed by MPS IIIB cases (80.6%) and less found in MPS IIIC or D cases (59.1%) (Fig. 4). This result agrees with Coutinho et al. publication; Sanfilippo A is the severe phenotype followed by Sanfilippo B while Sanfilippo C and D show milder symptoms [19].

**Fig. 3:**
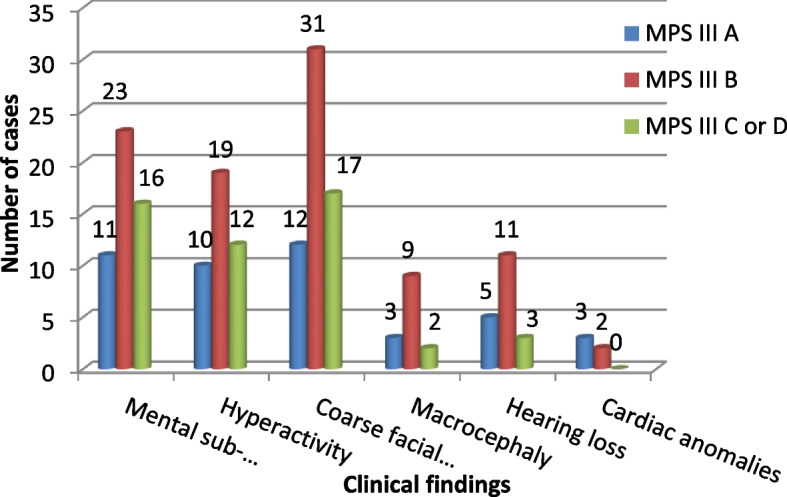
Signs and symptoms of the study group

**Fig. 4:**
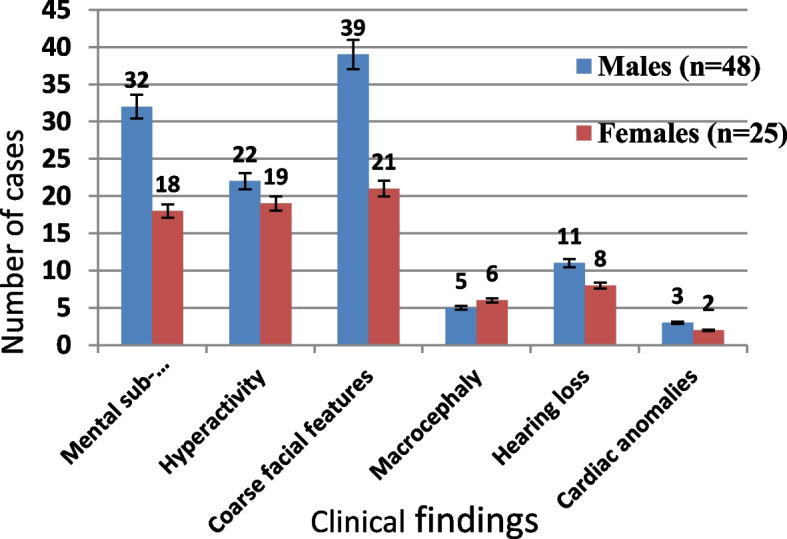
Signs and symptoms among different MPS III types

Abnormal facies (broad forehead, thick lips, wide-spaced teeth, and frontal bossing) were detected by clinical examination among of MPS III cases showed while the other 13 cases were younger to develop course facies (Figs. 3 and 4).

Poor documentation of auditory function in MPS III may be owing to the difficulty of the collection of audiometric data from patients with MPS III due to their behavioral problems [20]. Conductive issues also appear early in life, as otitis media has a reported rate of 91%, and tympanostomy tubes are placed in most patients before 5 years [21]. In the present study, general ear problems were found within 33.3% of MPS IIIA cases, 30.6% of MPS IIIB cases, and 13.6% of MPS IIIC or D cases. This differs from previously reported rates of hearing loss which are 100% (3/3) in MPS IIIB [21], 87% (48/55) in MPS IIIA [22], and 25% (1/4) in MPS IIID [23]. This may explain the point of view of Zafeiriou et al. in considering MPS IIIB as the severe form of all MPS III subtypes [21]. Jansen et al. result was more or less expected as MPS IIID is the mild form of Sanfilippo disease though it is the rare subtype [23].

MPS III is associated with cardiac anomalies as MPS I and II [24, 25]. The incidence of cardiac involvement in the present study is lower than that reported by Andrade et al. (60% of MPS IIIA cases, 30% of MPS IIIB cases, 4% of MPS IIIC cases, and 6% of MPS IIID cases had cardiac affection) [26]. Even though MPS III is the least MPS type that causes cardiac affection such as cardiomyopathy and valvular injury increases with age as aortic valve abnormality and mitral valve stenosis [18]. This may explain the lower cardiac affection in our MPS IIIA cases as we had few cases over the age of 10 years.

In the present study, urinary GAGs were high in 242 cases out of 535 referred patients, and 233 were diagnosed as MPS cases due to abnormal 2DE. Nine (3.7%) cases had high GAG level, but normal 2DE that occurs in many disorders with dysplasia [15, 27].

In Fateen (b) et al.’s study, the mean urinary GAG level for 43 MPS III patients was 34.4 mg/mmol creatinine. This is more or less similar to the mean urinary GAGs of our study MPS III urinary GAGs. The MPS III urinary GAG results were divided into 3 groups according to their age, 1–12 months, 1–5 years, and > 5 years, and their mean were 48.8, 47.4, and 27 mg/mmol creatinine, respectively (Table 2) [28]. Also, the mean of MPS IIIA cases urinary GAGs (25.6 mg/mmol creatinine) was lower than that of MPS IIIB cases (47.5 mg/mmol creatinine) (Table 3).

Seventy-three (73/233–31.3%) cases were diagnosed as MPS III due to the presence of heparan and heparan sulfate spots in 2DE. This is considered a quite high percentage when compared to other studies, MPS III formed 31.2% of all MPS cases in Turkia et al., 17.3% in Fateen(b) et al., and 28% in Fateen et al. [13, 15, 28]. Fateen (b) et al.’s study diagnosed 61 cases as Sanfilippo, and only 22 cases were diagnosed as MPS IIIB by enzyme assay while the rest of the cases remained undefined as MPS IIIA, C, or D due to the absence of MPS IIIA enzyme measuring method [28]. The same was found in Fateen et al. who diagnosed 134 MPS IIIB cases out of 177 cases of Sanfilippo and mentioned that the absence of MPS III A, C, and D was a limitation in their study [15].

Thirty-six (36/73–49.3%) patients had low alpha-*N*-acetylglucosaminidase enzyme activity and were diagnosed with MPS IIIB. Al-Sannaa et al.’s study reported 3/3–100% MPS IIIB cases, while 6/7–85.7% MPS IIIB cases were detected by Al-Jasmi et al.’s study and 22/61–36.1% MPS IIIB cases were diagnosed by Fateen et al. [11, 13, 26]. Meaning that according to our study, Al-Sannaa et al.’s study, Al-Jasmi et al.’s study, and Fateen et al.’s study, MPS IIIB is the commonest subtype of Sanfilippo followed by MPS IIIA through the Mediterranean region [15, 29, 30].

In the present study, 15/73–20.5% of patients had low *N*-sulphoglucosamine sulphohydrolase enzyme activity (MPS IIIA patients). No MPS IIIA was detected in some studies [29, 30], while in Estonia, all cases were MPS IIIA [31]. This could be explained by the high consanguinity rate among MPS IIIA patient (13/15 patients, 86.7%) cases accompanied by ethnic variations.

*N*-sulphoglucosamine sulphohydrolase enzyme activity was different than that of previously reported results in Egyptian cases. Cases with deficient MPS IIIA enzyme had a mean range of 0.25 ± 0.19 nmol/mg prot/17 h. Patients with normal MPS IIIA enzyme activity (MPS IIIC or D cases) had mean enzyme activity of 12.8 ± 0.23 nmol/mg prot/17 h. The range of normal cases is slightly higher in our study 3.4–42.6 nmol/mg prot/17 h. In comparison to Karpova et al.’s study, the normal range of this study was slightly higher than that of Karpova et al. The mean range of affected MPS IIIA patients in both studies is almost the same [32].

This could be explained by Egyptian ethnic variations. In the near future, molecular studies of MPS IIIA cases will provide more explanation about the difference in the enzyme level and phenotypic variations.

**Fig. 5:**
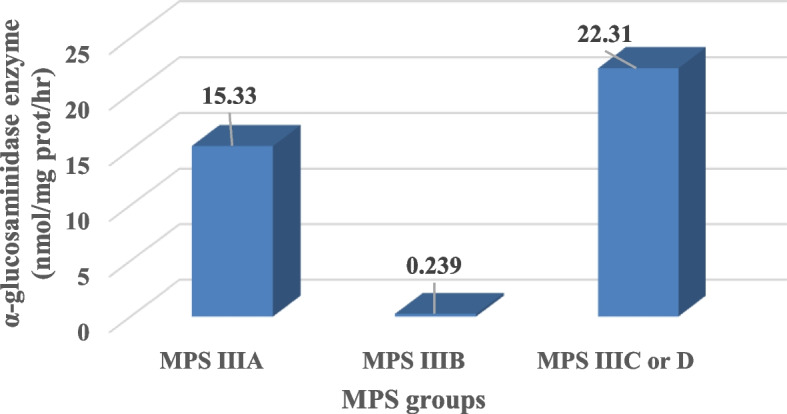
Comparison between the levels of α-glucosaminidase enzyme activity in three groups of MPS III: A, B, and C or D

When the results of alpha-*N*-acetylglucosaminidase enzyme activities for MPS III groups (A, B, and C or D) were compared, a significant difference was detected (Fig. 5). This characteristic difference was also found in Karpova et al.’s results [32]. This allows easy prediction of MPS IIIA cases whenever finding a case with high alpha-*N*-acetylglucosaminidase enzyme activities.

This study provided us with the easy, rapid diagnosis of MPS IIIA cases. Soon it will allow easy detection of the common Egyptian genetic mutations and its correlation with the phenotypic variation. MPS IIIA molecular study is now under search and very soon will allow molecular prenatal diagnosis via amniocentesis or cell-free DNA [18, 33]. Also, molecular studies may provide a seed for the promising MPS III cases of gene therapy [18].

MPS III has no enzyme replacement therapy (ERT) despite its availability for MPS I, II, IV, and VI as it is not able to bypass the blood–brain barrier and cure the neurological manifestations of Sanfilippo disease [15]. The availability of MPS III therapy either ERT or gene therapy will be an exciting milestone in encouraging the patients to seek diagnosis and raise the physician’s awareness.

## Conclusion

From this study, we concluded that MPS IIIA and B cannot be differentiated clinically as heparan sulfate accumulation causes almost the same symptoms in both types. Two-dimensional electrophoresis was a very good diagnostic tool for MPS type III in general, but the specific enzyme assay can differentiate and diagnose the four subtypes A, B, C, and D. Also, the mean age of diagnosis was 5 years, and most of the early diagnosed cases had previous family history of MPS III cases. More attention should be given to Sanfilippo disorder and its early diagnosis. MPS type III represents 31.3% of all MPS diagnosed cases in a period of 3 years of study, which is a very high percentage. We could notice a high incidence of MPS IIIA which was not diagnosed before as the only enzyme that could be measured for the last 25 years in Egypt was alpha-*N*-acetylglucosaminidase (MPS IIIB) enzyme activity. Out of MPS type III cases, 87.7% of them were to consanguine parents. Counseling of the families is an important aspect of the genetic specialist and our practice—to increase the awareness of the families and explain the course and nature of the disease. Prenatal diagnosis is offered to all diagnosed families as well as carrier detection to the other siblings. Awareness should be raised by the medics regarding the problems caused by consanguineous marriages: medical and genetic.

No enzyme replacement therapy is available yet for MPS type III as it is mainly a neurologic disorder; still, MPS IIIB is the commonest type in the Mediterranean region 36 (49.3%) cases versus 15 (20.6%) cases of MPS IIIA and 22 (30%) cases of MPS III types C or D.

### Supplementary Information

**Additional file 1.** Biochemical study.

## Data Availability

The article contains all the data generated during this study.

## Abbreviations

MPS: Mucopolysaccharidosis
MPS III: Mucopolysaccharidosis type III
GAGs: Glycosaminoglycans
2DE: Two-dimensional electrophoresis
HS: Heparan sulphate
CNS: Central nervous system
SGSH: *N*-Sulphoglucosamine sulphohydrolase
NAGLU: *N*-Acetyl-α-glucosaminidase
EDTA: Ethylene-di-amine-tetra-acetic acid
IEM: Inborn error of metabolism
ERT: Enzyme replacement therapy
