# Supplementary material for: Biochemical diagnosis of Sanfilippo disorder types A and B
Source: J Genet Eng Biotechnol. 2023 Nov 10;21:112. doi: 10.1186/s43141-023-00586-7 (PMC10638229; doi:10.1186/s43141-023-00586-7)
Supplement: Supplementary file 1 — Additional file 1. Biochemical study. [file 43141_2023_586_MOESM1_ESM.docx]

- **Biochemical study:**
- Sampling:
- 4 ml of urine were taken from each case. 1ml used to quantitate total GAGs level in urine and about 3 ml used to extract GAGs for electrophoresis.
- Five milliliter of venous blood sample were withdrawn from each person on Ethylenediaminetetraacetic acid (EDTA) tube and divided into 2 specimens:
- Two milliliters blood were centrifuged for 5 min at 2000 rpm to separate plasma used for MPS IIIB enzyme assay measuring and stored at -20 C° till use.
- Three milliliters blood were used to separate Leukocytes according to **cooper et al., 1988** **(14)** method and samples were stored at -20 C° until MPS IIIA enzyme assay was measured.
- GAGs quantitave measurement:

1. Total urinary GAGs were determined in samples taken from cases according to **de Jong et al., 1989** method **(11)**.
2. Two-dimensional electrophoresis test was done for GAGs extracted from urine taken from all cases and controls by **Applrgarth et al., 1997** method **(12)**.

Fluor metric measures:

- Fluorometric measurement of N-alpha-acetylglucosaminidase enzyme activity:

N-alpha-acetylglucosaminidase enzyme assay was measured for all cases with heparan and heparan sulfate spot in 2DE by **Tessitore et al., 2000 (13)**.

- Fluorometric measurement of N-sulphoglucosamine sulphohydrolase enzyme activity (measured for the first time in our lab, Biochemical Genetics Department, NRC):

MPS IIIA enzyme activity was measured according to **Karpova et al., 1996** method **(16)** to all MPS III patients with normal N-alpha-acetylglucosaminidase enzyme activity.

- Method:
- Ten microns of sample was added to 10 µl of substrate (4- Methylumbelliferone). Eppendorf was incubated for 17 hours at 47 °Ϲ.
- The reaction was stopped by adding Pi/ Ci buffer (Na- citrate added to Na-phosphate) and mixed well.
- α- Glucosidase was added and all samples were incubated for 24 hr at 37°Ϲ.
- The 2nd reaction was ended by adding 200 µl of carbonate stopping Buffer.
- Forty micromoles of 4-MU were added to 200 µl stop buffer to be used as a standard for calculations.
- The enzyme activity was calculated in relation to proteins content of each sample (measured by **Lowry et al., 1951** method) **(17)**.
- I Q test:

Fifty (68.5%) patients out of the 73 MPS III cases had Stanford-Binet IV Intelligence Test done.
